# Detection and quantification of C-terminally tagged proteins by in-gel fluorescence

**DOI:** 10.1038/s41598-024-66132-8

**Published:** 2024-07-08

**Authors:** Adrian C. D. Fuchs

**Affiliations:** Department of Protein Evolution, Max Planck Institute for Developmental Biology, 72076 Tübingen, Germany

**Keywords:** Fluorescence imaging, Biochemical assays

## Abstract

The analysis of recombinant proteins in complex solutions is often accomplished with tag-specific antibodies in western blots. Recently, I introduced an antibody-free alternative wherein tagged proteins are visualized directly within polyacrylamide gels. For this, I used the protein ligase Connectase to selectively attach fluorophores to target proteins possessing an N-terminal recognition sequence. In this study, I extend this methodology to encompass the detection and quantification of C-terminally tagged proteins. Similar to the N-terminal labeling method, this adapted procedure offers increased speed, heightened sensitivity, and an improved signal-to-noise ratio when compared to western blots. It also eliminates the need for sample-specific optimization, enables more consistent and precise quantifications, and uses freely available reagents. This study broadens the applicability of in-gel fluorescence detection methods and thereby facilitates research on recombinant proteins.

## Introduction

Western blots are the standard method for the detection of proteins in complex solutions^1^. However, their sensitivity, reliability and quantifiability vary greatly with the quality of the employed antibodies^2–6^. Therefore, when studying recombinant proteins, many researchers choose to introduce small protein tags, which can subsequently be detected with well-studied tag-specific antibodies^7–9^.

In a recent paper^10^, I developed an alternative method for the detection of tagged proteins. For this, I used the protein ligase Connectase to selectively fuse fluorophores to target proteins. Connectase is uniquely suited for this, as it offers a much higher specificity and catalytic efficiency compared to other protein ligases, such as Sortase A (see Supplementary Information for a detailed comparison)^11,12^. The labeled proteins were separated via SDS-PAGE and the fluorescent protein bands detected with an appropriate imager or fluorescence scanner. This method had significant advantages compared to western blots in respect of speed, sensitivity, signal-to-noise ratio, quantifiability, reproducibility and costs. However, for mechanistic reasons, it was limited to the detection of N-terminally tagged proteins.

**Figure 1:**
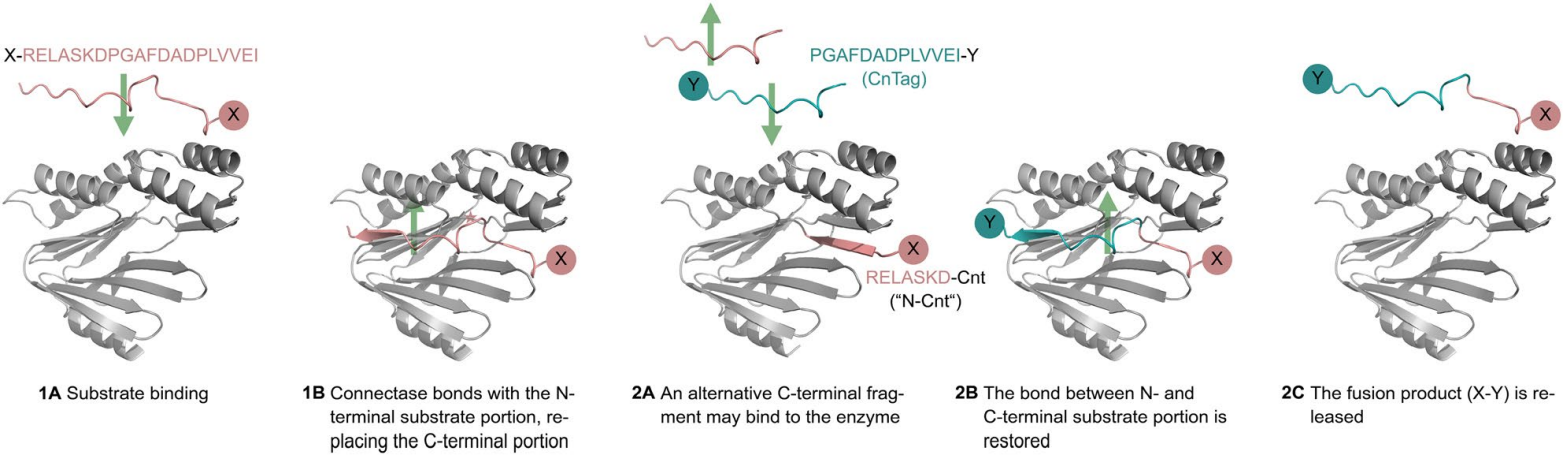
The Connectase reaction mechanism. Connectase binds to a 20 amino acid recognition sequence (red), which can be part of a peptide or a protein (symbolized as "X"; 1A). It forms an amide bond with the N-terminal portion of this sequence, while cleaving the C-terminal portion (1B). When a second substrate, which mimics the C-terminal portion (2A; cyan, with "Y" symbolizing a peptide or protein), is added to the reaction, it can be fused to the N-terminal portion (2B) to form a new fusion product (2C). The depicted structures were predicted with AlphaFold^13^. They may deviate from real structures.

Connectase acts on a 20 amino acid recognition sequence^11^. Upon binding (Fig. 1, step 1A)), it cleaves off the 13 C-terminal amino acids of this sequence ("CnTag"; step 1B). At the same time, Connectase (Cnt) forms a new amide bond between the first 7 residues of the recognition sequence and its own N-terminal amino group ("N-Cnt"). This reaction is reversible, meaning that the C-terminal (CnTag) fragment may be re-ligated to the N-terminal fragment. However, when a second substrate possessing the CnTag is added to the reaction, it can replace the original CnTag fragment to form a new fusion product (steps 2A-C).

**Figure 2:**
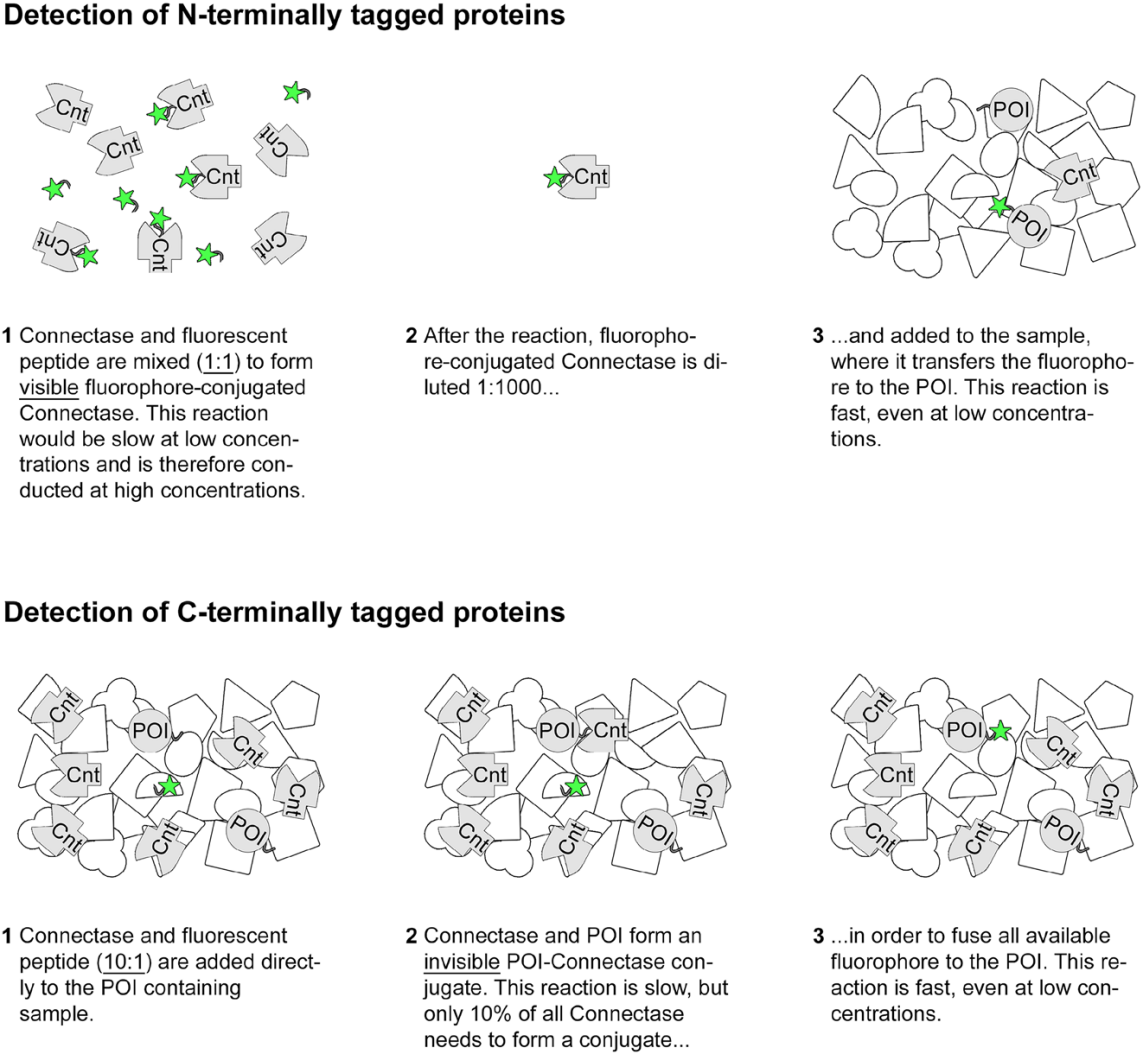
Differences between the methods for the detection of N- and C-terminally tagged proteins. N-terminally tagged proteins (top panel): Connectase (Cnt, grey forms) is mixed at a 1:1 ratio with peptides consisting of the Connectase recognition sequence and an N-terminal fluorophore (green stars). The fluorophore-Connectase conjugate is formed at a high concentration (1), diluted 1:1000 (2), and added to a complex sample (white forms) containing the protein of interest (POI, grey circles; 3). C-terminally tagged proteins (bottom panel): Connectase and fluorescent peptide are added directly to the POI containing sample, using a 10:1 ratio (1). Only 10% of all available Connectase enzymes need to form POI-Connectase conjugates (2), in order to fuse the POI to all available fluorophore (3).

The formation of N-Cnt is the rate-determining step (Fig. 1, step 1), whereas the formation of the fusion product (step 2) occurs much faster^11^. This was crucial for the development of a detection method for N-terminally tagged proteins^10^. For this, a peptide consisting of the Connectase recognition sequence and an N-terminal fluorophore was used. This peptide was incubated with Connectase to form the (fluorescent) N-Cnt intermediate. To speed up this rate-determining reaction step, relatively high concentrations of enzyme and reagent (5 µM each) were used (Fig. 2, top panel). As a result, an incubation time of 1 min proved sufficient. After that, the fluorophore-Connectase conjugate was diluted (1:1000) and added to a sample containing CnTagged protein of interest (POI). Here, it catalyzed the second, fast reaction step, the transfer of the fluorophore to the POI.

This strategy cannot be used for the detection of C-terminally tagged proteins with fluorescent peptides. Here, the rate-determining reaction step comprises the formation of a POI-Connectase conjugate. This step must be conducted within the POI containing sample. A separation of the two reaction steps, as described for the detection of N-terminally tagged proteins, is not possible. Hence, a C-terminal labeling approach was expected to be slow and inefficient at low POI concentrations. Furthermore, as the fluorescent peptide is not conjugated to Connectase in this case (Fig. 2), it could be more susceptible to proteases within the analyzed sample (e.g., a cell extract). In combination with longer reaction times, this could decrease the sensitivity of the assay.

In this paper, I solve these problems and present an efficient and accurate method for the detection and quantification of C-terminally tagged proteins. Crucial for this proved the employed Connectase: fluorophore ratio. For the N-terminal labeling method, a 1:1 ratio was optimal^10^. Here, it was not useful to add more Connectase, as this would have led to an increased amount of fluorophore-Connectase conjugate in the reaction equilibrium (see Fig. 1, all forms are in equilibrium). In other words, Connectase would have competed with POI for the available fluorophore. This would have decreased the fluorophore-POI signal and it would have led to a stronger fluorophore-Connectase signal. However, this problem is not relevant for C-terminal labeling, where no fluorophore-Connectase conjugate is formed. Consequently, a Connectase: fluorophore ratio of 10 : 1 can be used in this case. This has the advantage that only 10% of all Connectase molecules need to complete the first, rate-determining reaction step in order to use up all fluorophore in the second, fast reaction step. In the following, I show that this approach is as favorable as the method for the detection of N-terminally tagged proteins and therefore increases the applicability of the in-gel fluorescence method.

## Results and discussion

### Specific and sensitive protein detection

For this study, I used Connectase from *Methanosarcina mazei* and performed all reactions at room temperature (22 °C) in near-physiological buffer (pH 7, 150 mM NaCl, 50 mM KCl). I designed the peptide substrate PGAFDADPLVVEISEEGE-Cy5.5, consisting of the *Methanosarcina mazei* Connectase CnTag sequence, a 5 amino acid linker sequence (SEEGE; not required for labeling^11^), and the Cy5.5 fluorophore (Excitation: 683 nm / Emission: 703 nm). Furthermore, I fused the Connectase recognition sequence, RELASKDPGAFDADPLVVEI, without additional linker sequences to the C-termini of a representative range of target proteins (Maltose-Binding Protein (MBP)), Glutathione-S-Transferase (GST), Green Fluorescent Protein (GFP), Single domain Antibody (SdAb), Ubiquitin (Ub), Lysine-tRNA ligase (LysS), Heat shock protein 70 (Hsp70), as well as immunoglobulin G Light (LC) and Heavy Chains (HC).

**Figure 3:**
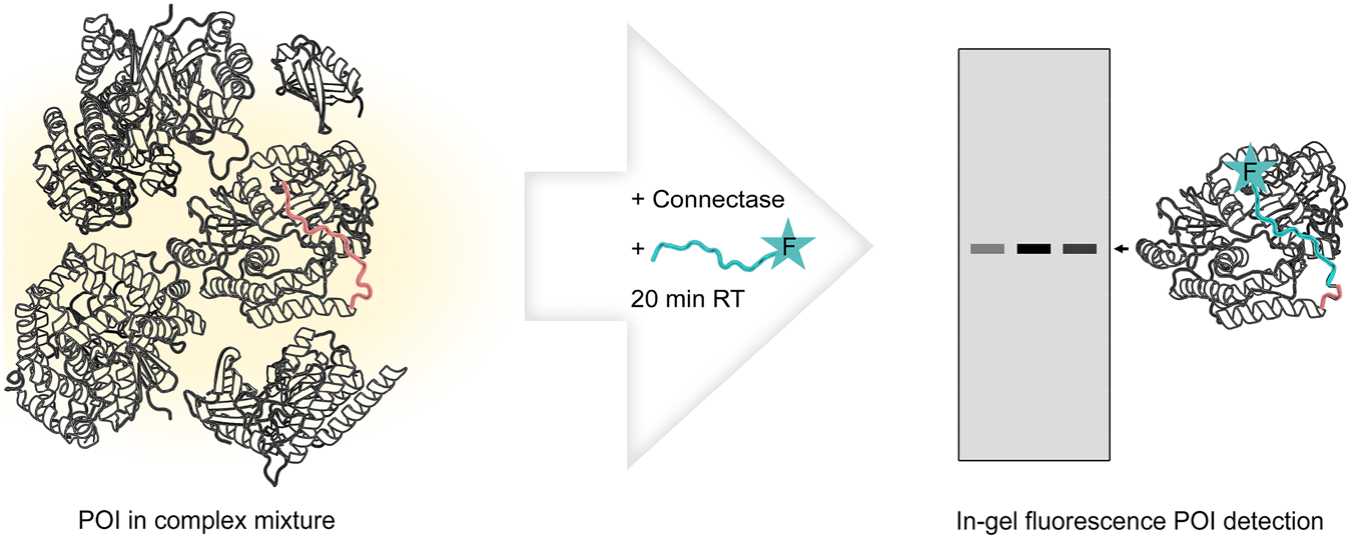
Assay protocol for the detection of proteins with C-terminal Connectase recognition sequence (red). A sample containing tagged protein of interest (POI, left) is incubated for 20 min with Connectase and fluorescent peptide (cyan). The POI can be visualized as a fluorescent band on a polyacrylamide gel (right).

Using these components, I performed all labeling reactions in this paper as follows (Fig. 3; a detailed protocol can be found in the Supplement):

1. Add 13.3 nM Connectase to the sample containing the tagged protein of interest.
2. Add 1.33 nM fluorescent peptide (see above) to the mixture
3. Incubate for 20 min at room temperature
4. Add ¼ vol. SDS-PAGE loading buffer, cook, and separate via SDS-PAGE
5. Image on a suitable fluorescence imager

**Figure 4:**
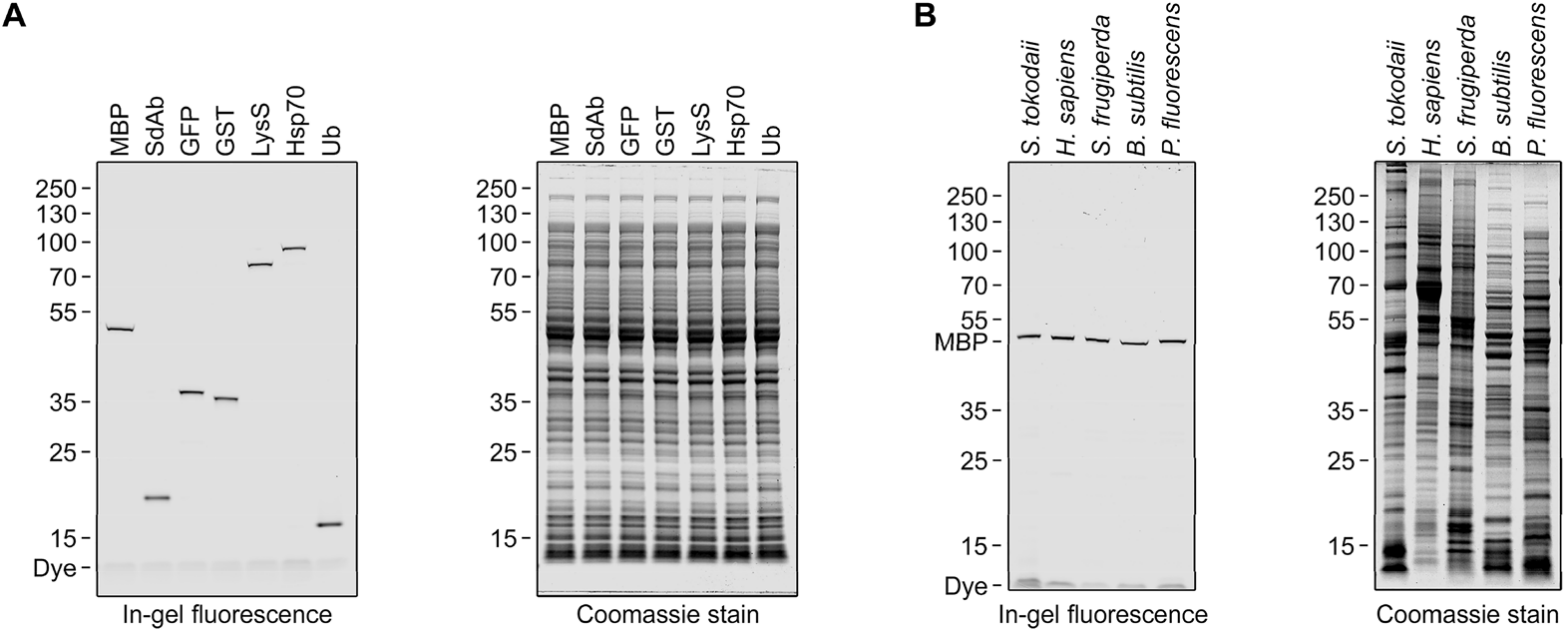
Detection of different proteins in *E. coli* cell extract (**A**) and detection of a representative protein (MBP) in different cell extracts (**B**). (**A**) Various proteins (indicated; 300 fmol per lane) with a C-terminal Connectase recognition sequence were mixed with *E. coli* cell extract (20 µg protein per lane) and detected as described in the main text (see general protocol). Shown are SDS-PAGE fluorescence scans before (left) and after (right) Coomassie staining. Unconjugated fluorescent peptide (“Dye”, 2.5 kDa) is seen at the bottom of the left gel (see also Figure S1). (**B**) C-terminally tagged MBP (300 fmol per lane) was mixed with various cell extracts (indicated; 20 µg protein per lane) and visualized as in (**A**).

In a first experiment (Fig. 4A), I analyzed samples containing 20 µg *Escherichia coli* cell extract protein and 300 fmol of each protein of interest (corresponds to 3–21 ng target protein, depending on the molecular weight). All proteins were detected as clean and sharp bands with a good signal-to-noise ratio. Residual fluorescent peptide is visible as a weak band slightly above the bromophenol blue dye running front, indicating an almost complete transfer to the target proteins. Similar results were obtained in experiments with various eukaryotic (*Spodoptera frugiperda* Sf9 cells, Human embryonic kidney HEK293 cells), bacterial (*Pseudomonas fluorescens*, *Bacillus subtilis*) or archaeal (*Sulfolobus tokadaii*) cell extracts (Fig. 4B). This shows the general suitability of the method for the detection of target proteins in complex solutions.

**Figure 5:**
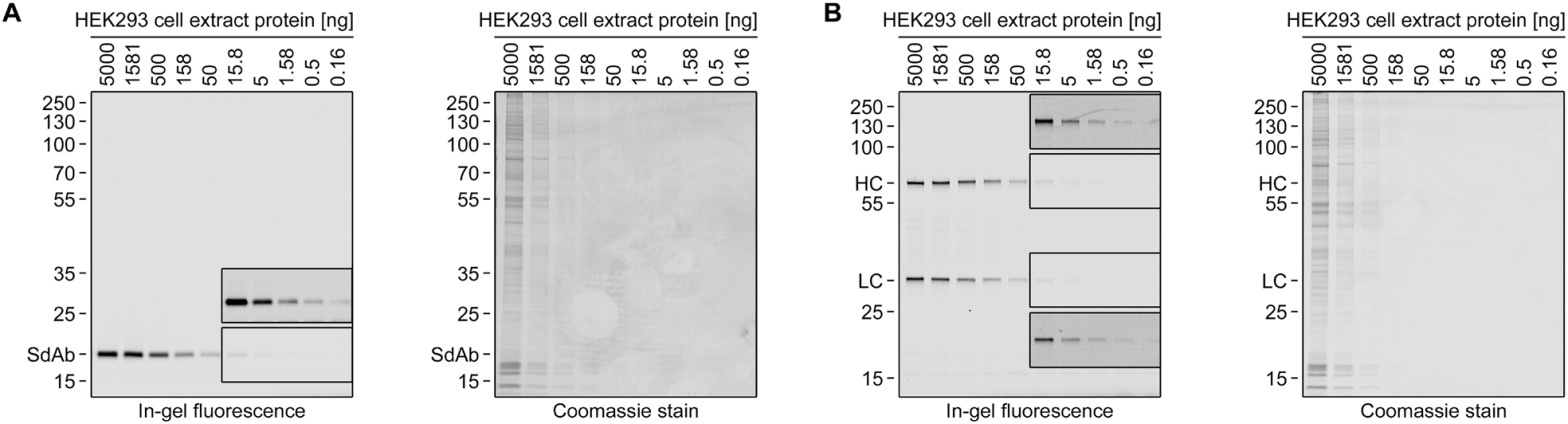
Detection of tagged proteins expressed in mammalian cells. C-terminally tagged single domain antibody (SdAb, **A**) or immunoglobulin G light (LC, **B**) and heavy chains (HC) were expressed in HEK293 cells. The cells were lysed and the protein content of the lysate determined. A serial dilution of the lysate was used for in-gel fluorescence detection (left gel) and subsequent Coomassie staining (right gel). The insets show the same area of the gel with increased contrast. This representation was chosen as some information (i.e., either the difference between intense bands or the signal of faint bands) in the original image (16 bit or 65,536 shades of grey) is otherwise poorly visible in the Figure. (8 bit or 256 shades of grey).

To test the method for proteins expressed in mammalian cells, I produced tagged single domain antibody as well as tagged immunoglobulin G heavy and light chains in HEK293 cells. The cells were harvested, lysed, and the labeling performed right within the lysis buffer (RIPA buffer, containing 0.1% SDS, 0.5% sodium deoxycholate and 1% NP-40). On the resulting gels (Fig. 5), all proteins were detected with good sensitivity (< 1 ng cell extract protein) and without significant background. By comparison, a western blot experiment conducted in a previous study^10^, encompassing the same expression protocol and instrumentation, as well as a comparable fluorophore (IRDye 680), required > 500 ng HEK293 cell extract protein for POI detection.

**Figure 6:**
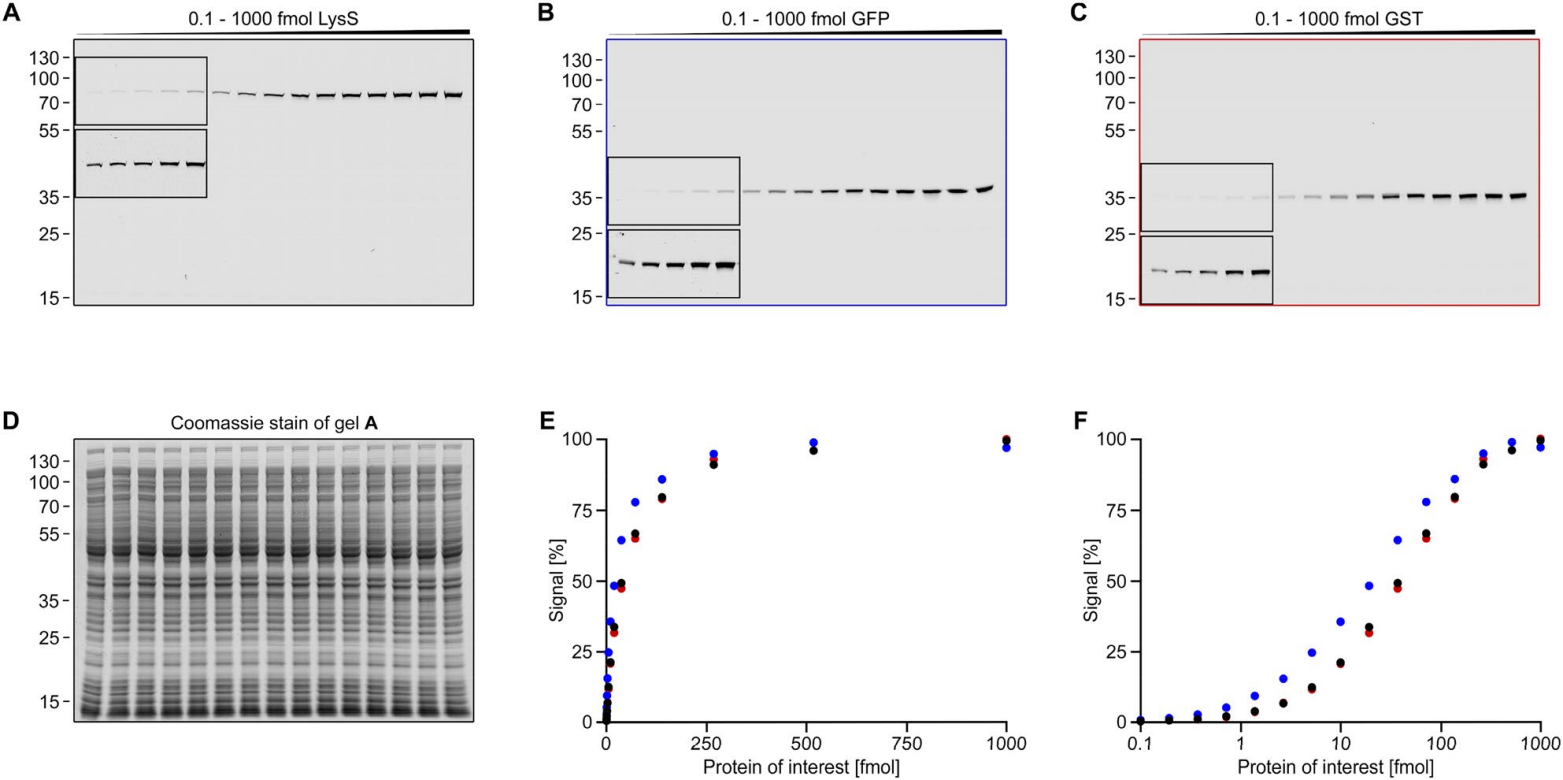
Signal-substrate relationship of in-gel fluorescence assays. A serial dilution of three proteins, LysS (**A**), GFP (**B**), and GST (**C**), was prepared in *E. coli* cell extract (20 µg protein per lane). The proteins were detected via in-gel fluorescence (**A**-**C**; the insets show the same area of the gel with increased contrast) and Coomassie stains of the gels prepared (see source data file; **D** shows one representative gel). The band signals were determined densitometrically and plotted against the protein quantities on a linear (**E**) or logarithmic (**F**) scale. LysS signals are represented as black dots, GFP signals as blue dots, and GST signals as red dots.

To analyze the signal-to-substrate relationship in more detail, I mixed different quantities (0.1–1000 fmol) of purified tagged proteins with 20 µg *Escherichia coli* cell extract protein (Fig. 6 and Figure S1). The resulting gels show a good signal-to-noise ratio, even at very low target protein quantities (1–7 pg in the first lane with a > 10^6^ excess of cell extract protein). The fluorescent peptide substrate migrates at the bottom of the gels (see Figure S1A; not visible in Fig. 6), slightly above bromophenol blue in typical SDS-PAGE loading buffers. Thus, for the detection of small proteins, a gel system with good separation in this region should be used. The intensity of the fluorescent peptide band diminishes gradually with higher POI quantities, indicating its near-complete conjugation to the target protein.

To obtain signal-to-substrate curves, I performed densitometric analyses of the target protein bands and plotted the values against the molar quantities. The resulting plots show hyperbolic curves on a linear scale (Fig. 6E) and sigmoidal curves on a logarithmic scale (Fig. 6F). The detection limit is seen in all cases at 0.1 fmol, the half-maximum signal at 20–40 fmol, and the maximum signal at > 300 fmol target protein.

This sensitivity and specificity is not achieved with typical western blots. Although they vary widely, most western blots have a detection limit in the range of roughly 1–1000 fmol and often show a higher background. Examples for well-prepared blots can be found in publications of Janes^14^, who detected as little as 7 fmol (i.e., 0.5 ng) p38 and 17 fmol ERK2; Butler et al.^15^, analysing ENPP1 down to 26 fmol and Fam3a (500 fmol); Deng et al.^16^, studying Smad3 (50 fmol); Mathews et al.^17^, visualizing Fetuin-A (833 fmol); and Wang et al.^18^, detecting hdm2, hdmx and p53 (8–16 fmol). Consistent with these findings, a previous investigation^10^ revealed a detection limit of approximately 100 fmol in a Western blot experiment. This experiment was conducted under conditions comparable to those in Fig. 6 and S1, encompassing identical instrumentation, cMyc-tagged proteins (GST, SdAb, as in Figure S1), and a similar fluorophore (IRDye 680).

However, the obtained hyperbolic signal-to-substrate curves (Fig. 6E) are not suitable for quantifications. Here, linear relationships are preferred. Although western blot signal-to-substrate curves are complex and affected by a multitude of parameters, they often assume a much broader sigmoidal shape, in which a close-to-linear range can be identified^14,19,20^. This broader curve is caused by the use of far more fluorophore in typical western blots (2—100 pmol antibodies according to manufacturer instructions) compared to the in-gel fluorescence method (5 fmol per gel lane). Consequently, the maximum signal, where all fluorophore is conjugated to the target protein is reached at lower target protein concentrations in in-gel fluorescence assays (~ 300 fmol in Fig. 6). While this prevents western blot like quantifications, it brings many advantages for protein detection. The amount of fluorophore (a maximum of 5 fmol per band) is well-suited for common fluorescence imagers. It is high enough to make use of their broad dynamic range, but low enough to ensure clean and sharp bands without unspecific binding. In addition, fluorophore limitation enables the use of a different and far more accurate quantification strategy, which will be discussed in the next section.

### Relative and absolute protein quantifications

When two proteins with C-terminal Connectase recognition sequences are present within the same sample in sufficient quantity (> 300 fmol in total), they should compete for the limited fluorophore. The signal ratio of the two resulting gel bands can be expected to depend on the relative quantity of both proteins, their relative reactivity, and the relative brightness of each protein-fluorophore conjugate. This idea can be summarized as

1 $$Signal\;ratio_{A/B} = \frac{Reactivity\;Protein\;A}{{Reactivity\;Protein\;B}}\;x\; \frac{Brightness\;Protein\;A-Fluo.}{{Brightness\;Protein\;B-Fluo.}}\;x\; \frac{Quantity\;Protein\;A}{{Quantity\;Protein\;B}}$$

One could hypothesize that relative reactivity and brightness remain almost constant with increasing protein quantities. In that case, both factors could be summarized in the constant k_A/B_:

2 $$Signal\;ratio_{A/B} = {\text{k}}_{A/B}\;x\;\frac{Quantity\;Protein\;A}{{Quantity\;Protein\;B}}$$

**Figure 7:**
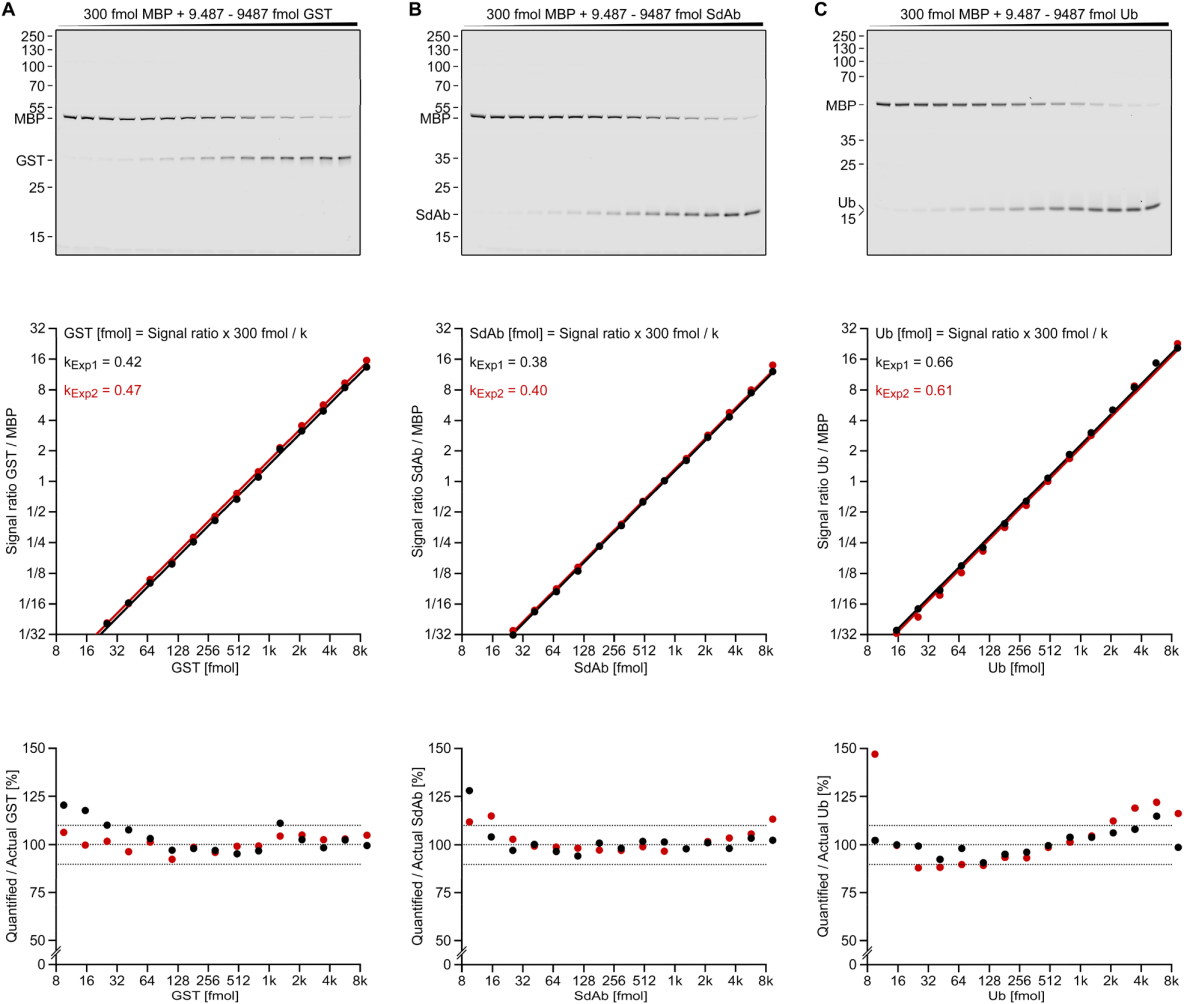
Competition assays with constant reference protein quantities enable accurate POI quantifications. Serial dilutions of three C-terminally tagged proteins, GST (**A**), SdAb (**B**) and Ub (**C**), were prepared in two independent experiments (n = 2). They were mixed with constant quantities of a tagged reference protein (300 fmol MBP) in *E. coli* cell extract (20 µg per lane). The samples were analyzed via in-gel fluorescence (top layer; only one representative gel (Experiment 1) is shown for each POI). The signal ratio (POI signal/reference signal) was determined densitometrically and plotted against the POI quantities (middle layer). For each data series (Exp.1 (black) and Exp. 2 (red)), a linear data fit (y = k*x, see Eq. 2) with slope k was created. The deviation of the actual data from these data fits are analyzed on a linear y-axis scale in separate plots (bottom layer). Consequently, these plots show which errors are made when quantifying samples using Equations. (2 or 3).

To test whether this equation can describe experimental data, I designed experiments with increasing quantities (9.607–9607 fmol) of one protein (POI) and constant quantities (300 fmol) of a reference protein (Ref; Fig. 7 and Figure S2). After labeling and SDS-PAGE analysis, the fluorescent bands were quantified densitometrically. Finally, the signal ratio (i.e., signal POI band / signal reference band) was plotted against the POI quantities.

The obtained data points in each experiment show an almost perfect linear relationship between signal ratio and POI quantities. The data is best approximated with a fit in the form of y = k * x or signal ratio = k_POI/Ref_ * [POI] / [Ref], suggesting the general validity of (Eq. 2). This data fit can therefore be used to determine k_POI/Ref_, which is seen as a Y-shift of the curves on a logarithmic scale (Fig. 7, middle layer) and as a different slope on a linear scale (not shown).

**Figure 8:**
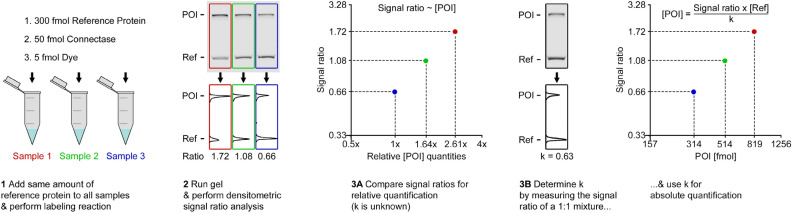
How to quantify proteins with in-gel fluorescence competition assays. The reaction is performed by adding equal quantities of reference protein (300 fmol), Connectase (50 fmol), and fluorescent peptide (5 fmol) to all samples (1). The samples are then separated by SDS-PAGE and the signal ratios between POI and reference bands determined (2). A comparison of the signal ratios is sufficient to determine relative POI quantities in the samples (3A; Eq. 3). When a sample with a known POI concentration is available, it can be used to determine the factor k, which enables absolute POI quantifications (3B; Eq. 4).

Taken together, this means that similar experiments can generally be used for protein quantification. For this, samples with unknown POI quantities need to mixed with 300 fmol of reference protein and analyzed as described above (Fig. 8, step 1). The relative POI quantities in each sample can then be determined by comparing the resulting signal ratios (step 2) according to:

3 $$\left[ {POI} \right] \sim Signal\;ratio\quad {\text{| Relative}}\;{\text{Quantification}}\;{\text{(step}}\;{\text{3A)}}$$

For example, a 1.64 × higher signal ratio in one sample compared to another indicates 1.64 × more POI (see green and blue samples in Fig. 8). Note that k_POI/Ref_ does not need to be known for this. When at least one sample with known POI concentration is available, k_POI/Ref_ can be determined with Eq. (2). It can subsequently be used for the absolute (i.e., “how many fmol?”) quantification of other samples with

4 $$\left[ {POI} \right] = \frac{Signal\;ratio}{{{\text{k}}_{A/B} }}\;x\;\left[ {Ref} \right]\quad {\text{| Absolute}}\;{\text{Quantification}}\;{\text{(step}}\;{\text{3B)}}$$

### Accuracy, reproducibility and comparability of results

In order to analyze the average error in such quantifications, I generated an additional set of plots (Fig. 7, bottom layer). For this, I used Eq. (4) to quantify POI values and compared them to the POI quantities that were actually used in the experiment (see Y-axis). Particularly accurate values were obtained when similarly intense POI and reference bands were compared, while higher errors were seen when very different bands were compared (on the left or rightmost side of the gels). Here, small errors in the quantification of the less intense band lead to high errors in the calculated POI quantities. Based on this, I propose to use the method for signal ratios between 0.05 and 20, where the average error across all experiments amounts to 4.7%. This is sufficient to quantify samples with a 400 × difference in POI levels.

By comparison, quantitative western blots usually have only a near-linear signal-to-substrate relationship over a narrower range. The band signals deviate more from standard curves, resulting in higher quantification errors. Examples for well-executed western blot quantifications can be found in publications of Pillai-Kastoori et al.^20^, who observed near-linear signal-substrate curves in several experiments with 1.25–30 µg cell extract; Butler et al.^15^, who analyzed many samples containing 0.16–40 µg cell extract and observed linearity in certain subranges, depending on the target protein, the detection method, and experimental replicate; Janes^14^, who presents examples for hyperbolic and linear curves for extracts ranging from 1 to 50 µg, as well as hyperbolic curves for purified proteins ranging from 0.5 to 63 ng; and others^19,21–23^. Consistent with these findings, a previous investigation^10^ revealed a linear range between 1000 and 12,500 fmol for Hsp20 and SdAb (shown in Fig. 7B) and 400–12,500 fmol for Ubiquitin-activating enzyme E1, with an average deviation of 15.9% from linear data fits. This experiment was conducted under conditions comparable to those in Fig. 7, encompassing identical instrumentation and a similar fluorophore (IRDye 680).

Overall, I conclude that if deviations from the linear relationship in Eqs. (3 and 4) exist, they are small enough to warrant sufficiently accurate quantifications. Furthermore, the results of replicate experiments on different gels are similar enough to enable the comparison of samples on different gels in certain cases. For this, however, it is essential that both experiments were performed with exactly the same reaction parameters (i.e., labeling temperature, time and buffer). Any difference in these parameters can be expected to change the relative reactivity in Eq. (1), and with it k_POI/Ref_, so that both experiments become incomparable.

### Applicability of the method

The assays presented so far characterize the basic features of the detection method, but they differ from real applications in the lab. As a representative example for such an application, I chose a yet unpublished case study, in which I aimed to use Connectase as a protein ligase^11^ to generate homogeneous antibody conjugates. To this end, I designed αHER2 light and heavy chains with a C-terminal Connectase recognition sequence plus a C-terminal Strep-tag. The idea was to use the Strep-tag for purification and to replace it afterwards, by conjugation of the heavy or light chain C-termini to the target molecules. The antibodies should be produced in adherent HEK293 cells. Without experience in this approach, I wondered how many cell culture plates I would need to obtain a quantity of 1 mg and for how long transiently transfected cells would produce it. To answer these questions, I transfected cells on a 75 cm^2^ plate and exchanged the cell culture medium (10 ml) on 10 successive days. Then, I used 2.5 µl of each cell culture supernatant, added 300 fmol reference protein, and performed the labeling reaction.

**Figure 9:**
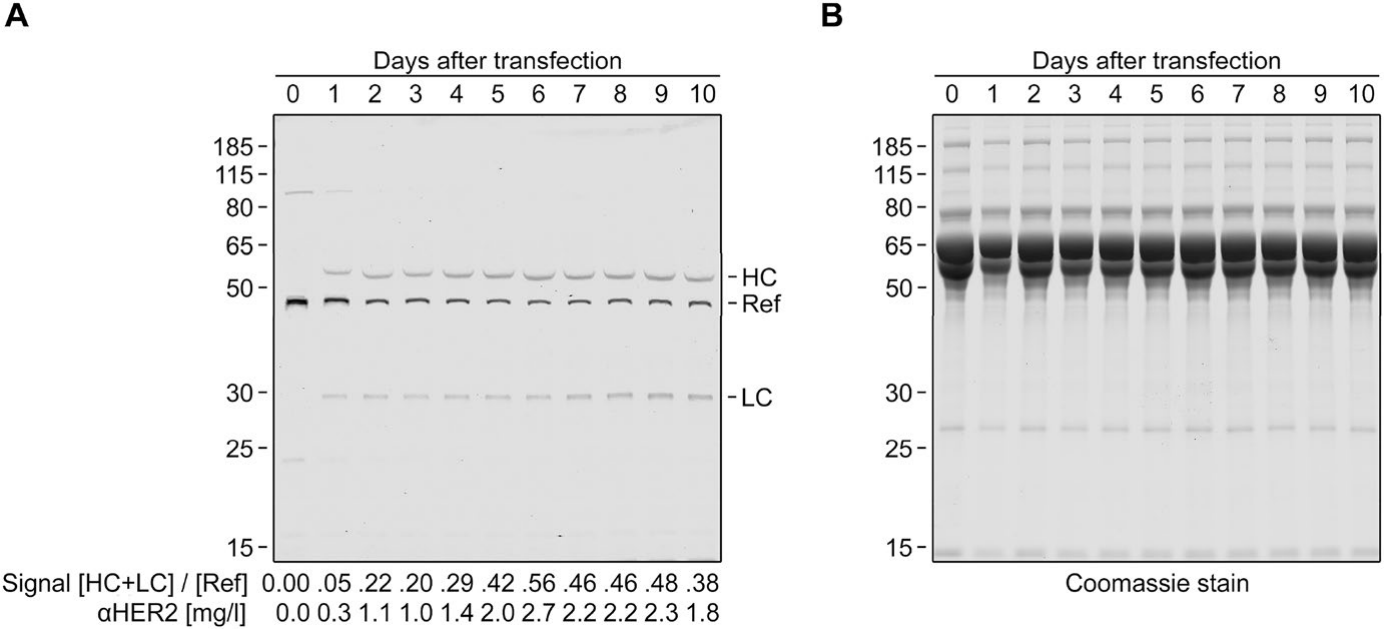
In-gel fluorescence analysis of antibody expression (**A**) and Coomassie stain of the same gel (**B**). HEK293 cells were transfected with plasmids encoding for antibody light (LC) and heavy chains (HC) with C-terminal Connectase recognition sequences. The cell culture supernatant was exchanged every day. A fraction of each supernatant was mixed with 300 fmol reference protein (Ref) and analysed by in-gel fluorescence (**A**). The signal ratio [HC + LC]/[Ref] is shown at the bottom of the gel. It was used to determine the antibody concentration in each sample. A Coomassie stain of the same gel (**B**) shows the cell culture medium constituents.

The resulting gel (Fig. 9) shows that antibody production per day gradually increases over the first six days and still persists after ten days. This suggests that the transfected cells retain the plasmids and that the plasmids are inherited on cell division. Without knowledge of k, an exact absolute antibody quantification is not possible, but a rough estimate can be made by assuming k = 1. With this, I expected a yield of 0.17 mg antibody per 75 cm^2^ plate in ten days. Based on this estimate, I used ten such plates for antibody production and eventually obtained 1.32 pure antibody. In accordance, the pure antibody could be used to obtain the actual k value, k = 1.03 (Figure S3).

Taken together, the example shows that the method is robust and specific enough to detect target proteins, right within complex cell culture medium. It shows how useful estimates for target protein quantities can be made, even without knowledge of k. Finally, it highlights the synergy between different Connectase-based methods, as the recognition sequence to generate antibody conjugates could also be used for protein detection.

## Conclusion

The C-terminal labeling method presented here can be used in a similar way as the originally proposed N-terminal labeling method^10^. The main differences are the use of a different tag sequence on the protein of interest, a different fluorescent peptide substrate, and a different assay protocol (different Connectase and fluorophore concentrations, different incubation times).

Taken together, both methods present an attractive way to detect and quantify recombinant proteins in complex solutions. Compared to the current state of the art for quantitation, fluorescent western blots^14,20^, in-gel fluorescence assays require much shorter hands-on and incubation times. The assay protocol is always the same and does not need to be adapted to the protein type or different concentrations. Gels can be used for subsequent analysis, for example Coomassie staining. The obtained sensitivity is often > 100 × higher and the signal-to-noise ratio is usually better. Quantifications are more accurate and can be performed over a wider linear range. Finally, the reagents are made freely available in assay kits (see materials and availability).

As of now, the main limitation of the technology is its requirement for uncommon protein tags. Currently, these tags can only be used for protein ligations^11^ and in-gel fluorescence. By contrast, some tags used for western blotting, such as the Strep-tag, can be used more widely, for example in pulldowns or protein purification. Furthermore, a wide range of specific materials, from antibodies to Streptavidin-conjugated beads or Biotin blocking kits are available in this case. While it is unclear, whether Connectase tags can be developed to be as versatile as the Strep-tag, it is clear that we are only starting to uncover the potential of this technology. It is conceivable that it also proves useful in other protein detection formats, such as microplate assays, cytometry or fluorescence microscopy. I also envision its use in site-specific immobilization, protein purification, or preparative protein conjugation. With the realization of some of these options, Connectase tags may become more wide-spread and versatile. For now, I believe to have developed a useful tool for researchers, which currently use western blots as a main method to study their recombinant proteins. For these colleagues, it may be worthwhile to introduce Connectase tags in their proteins in order to save time and money or to increase sensitivity, accuracy and reproducibility.

## Methods

### Cloning, expression and purification

The sequences of all proteins in this study are listed in Dataset S1. The genes were synthesized (Biocat, Heidelberg) or produced via PCR using optimized codon frequencies (*E. coli* or human). They were cloned into the pET30b( +) vector (restriction sites: NdeI, XhoI) for expression in *E. coli* or the pcDNA3.1 vector (restriction sites: HindIII, XhoI) for expression in HEK293 cells.

For recombinant expression in *E. coli*, BL21 gold cells were transfected with the respective plasmids and grown in lysogeny broth medium with 50 µg/l kanamycin at 22°C. Protein expression was induced at an optical density of 0.4 at 600 nm with 500 µM isopropyl-β-D-thiogalactoside. Cells expressing soluble proteins were harvested after 16 h, resuspended in buffer (100 mM Tris–HCl, 5 mM MgCl_2_, 1 × c0mplete EDTA-free protease inhibitor cocktail (Roche), 0.02 g/l DNAse, pH 8.0), lysed by French press, and cleared from cell debris by ultracentrifugation (120,000*g*, 45 min, 4 °C).

For recombinant expression in human cells, HEK293 cells were cultured in 6 well plates with DMEM medium at 37°C. At 70% confluence, the cells were transfected with 2500 ng of the respective plasmids using 12.5 µl Lipofectamine 2000. The cells were harvested after 24h.

For protein purification, soluble His_6_-tagged proteins were applied to HisTrap HP columns (20 mM Tris–HCl pH 8.0, 250 mM NaCl, 20—250 mM imidazole; all columns were obtained from Cytiva) and, in a second step to a Superdex 75 size-exclusion column (20 mM HEPES–NaOH pH 7.5, 100 mM NaCl, 50 mM KCl, 0.5 mM TCEP). All chromatography steps were performed on an Äkta Purifier FPLC (GE Healthcare) using Unicorn v5.1.0 software. Purified proteins were supplemented with 15% glycerol, flash frozen in liquid nitrogen and stored at -80°C.

### In-gel fluorescence assays

Generally, all reactions were conducted as described in the assay protocol (Supplemental Data). Specifically, samples were prepared in buffer A (50 mM sodium acetate, 50 mM MES-NaOH, 50 mM HEPES–NaOH, 50 mM KCl, 150 mM NaCl, 0.5% NP-40, pH 7.0) or RIPA buffer (50 mM Tris–HCl, 150 mM NaCl, 1% NP-40, 0.5% deoxycholate, 0.1% SDS, pH 7.5). To avoid protein loss, PCR tubes were used for their reduced surface area. Where sample impurities should be simulated (Figs. 4, 6, 7, S1, and S2), 6 g/l *E. coli* cell extract protein (determined with the bicinchoninic acid (BCA) method) were added.

The labeling stock solution was prepared by mixing 5 µM PGAFDADPLVVEISEEGE-Cy5.5 reagent (synthesized by Intavis; TFA salt; HPLC purity 97.85%; molecular weight (2565.27) confirmed via NMR) with 50 µM Connectase in buffer A. This solution was then diluted 1250 × in buffer A and mixed with the sample to be analyzed (ratio 1:2 for a final concentration of 1.33 nM reagent and 13.33 nM Connectase). The reactions were incubated for 20 min at room temperature and stopped with 1/4 vol. SDS loading buffer (250 mM Tris, 8% SDS, 0.1% bromophenol blue, 40% glycerol, pH 6.8). The samples were heat incubated (95°C, 3 min) and separated (5 µl loading volume) with mPAGE 12% BisTris gels (Merck; 200 V constant; general running buffer: 50 mM MOPS, 50 mM Tris, 1 mM EDTA, 0.1% SDS, pH 7.7; running buffer for Ubiquitin gels (Figs. 7C and S1A): 50 mM MES, 50 mM Tris, 1 mM EDTA, 0.1% SDS, pH 7.3). The gels were imaged immediately after the run with an Odyssey CLx fluorescence scanner (Licor; 700 nm channel, Intensity: 6.5, focus offset: 0.5 mm, resolution: 42 µm, quality: lowest). 16 bit images containing all fluorescence data were generated with the instrument software, exported, and analyzed with Image studio 5.3 or ImageJ 1.52a using the Bio-Formats 6.11.0 plugin. For quantifications, the signal of the whole band, including potential smears or shadows was used. For a delayed/later analysis, the gels were stored in fixation solution (50% methanol, 10% acetate, at 4°C in the dark; no maximum fixation time). In most cases (Figs. 4, 5, 6, 9 and S1), gels were stained after the fluorescence scan with Coomassie solution (5.8% H_3_PO_4_, 10% (NH_4_)_2_SO_4_, 0.12% Coomassie G-250, 20% Ethanol, 5% Methanol), de-stained (10% acetate), and re-imaged with the fluorescence scanner (same settings, except for automatic intensity). Details on sample preparation and other experiment-specific information are provided in the following.

For the preparation of Fig. 4A, equal quantities of C-terminally tagged proteins (300 fmol MBP, SdAb, GFP, GST, LysS, Hsp70, Ub) were used in the labeling reaction (see above). For Fig. 4B, various cell extracts were prepared. *Spodoptera frugiperda* SF9 and HEK293 cells were gifts from Dr. Birte Hernandez Alvarez; *Pseudomonas fluorescens* and *Bacillus subtilis* were cultured in LB at 30°C up to OD 1.5; *Sulfolobus tokodaii* cells were a gift from Dr. Jörg Martin. All cells were harvested by centrifugation, resuspended in RIPA buffer (supplemented with 0.02 g/l DNAse, c0mplete protease inhibitor, 5 mM MgCl_2_ and 5 mM TCEP), lysed by sonication, and centrifuged again to obtain the soluble cell extract fraction. The protein concentration was determined with the *BCA* method. Finally, 120 nM tagged MBP was mixed with 6 g/l of each cell extract and visualized as described in the general procedure (see above).

For Fig. 5, HEK293 cells expressing C-terminally tagged proteins (SdAb (A); αHER2 IgG light and heavy chains (B)) were harvested and lysed in RIPA buffer. The protein concentration was determined with BCA and a 3.162x (i.e., √10) serial dilution, starting with 2 g/l, was prepared. All samples were labeled with 1/2 vol. labeling solution and visualized (see above).

For Fig. 6 and Figure S1, a serial dilution of six individual C-terminally labeled proteins (LysS, GFP, GST (Fig. 6); Ubiquitin, MBP, SdAb (Figure S1)) was generated, ranging from 400 nM to 40 pM (1.9307 × steps). The samples were mixed with 1/2 vol. labeling solution and visualized (see above). Two independent replicates were prepared for each experiment. For analysis, the average signal of the 400 nM samples, corresponding to 125 fmol on the gel, were set as 100% signal.

For Fig. 7 and Figure S2, two replicates of five competition experiments were performed. In each case, 1 vol. of a reference protein solution (240 nM MBP) was mixed with 1 vol. of protein of interest solution (7.59–7590 nM GST, SdAb, Ub (Fig. 7); LysS, GFP (Figure S2)), labeled with 1 vol. of labeling solution, and visualized as described. The signal ratio between reference and protein of interest band intensities was determined with Image Studio 5.2 and plotted against the protein of interest quantities (middle layer). k values were determined for signal ratio 0.1—10 based on Eq. (4) ([POI] = Signal ratio x [Ref] / k). The average of these values is shown in the chart and visualized as a linear curve. The percentile deviation between the POI quantities calculated with Eq. (4) and the actually used POI quantities was plotted against the (actual) POI quantities (lower layer).

For Fig. 9, HEK293 cells were grown in a 75 cm^2^ flask to 70% confluency, using Dulbecco's Modified Eagle Medium (DMEM) with fetal calf serum. The cells were transfected with lipofectamine 2000 (Thermo), according to the manufacturer's instructions (47 µl Lipofectamine 2000, 55 µg of each plasmid). The cells were grown for ten days (no "splitting") and the medium was exchanged every day. The collected medium samples were centrifuged (1 min, 10,000*g*). 2.5 µl of each supernatant were mixed with 300 fmol C-terminally tagged MBP and visualized as described in the general procedure (see above).

### AlphaFold models

For Fig. 1, an AlphaFold2 model of a complex between *M. mazei* Connectase and *M. mazei* recognition sequence (RELASKDPGAFDADPLVVEI) was generated. This was done by gathering and aligning all available Connectase sequences from *Methanosarcinales* and *Methanomicrobiales*. The sequences were trimmed to the length of the *M. mazei* variant. A GSGSGSG linker was added to the C-termini, as well as a region of the MtrA sequence from each organism. This region included the KDPGA motif and 15 residues N- and C-terminal of that motif. The structure of the *M. mazei* sequence within this dataset was then predicted with AlphaFold2, using the other sequences as a custom alignment for the process. For this, the Colab AlphaFold implementation with Jackhmmer available under https://colab.research.google.com/github/sokrypton/ColabFold/blob/main/beta/AlphaFold_wJackhmmer.ipynb was used with standard settings. The best-scoring model from this prediction was in good agreement with the known co-structure of the respective *M. jannaschii* complex (PDB 6ZW0). It is shown in Fig. 1 (step 2 and 4), without the linker and without 10 residues N-terminal of said MtrA region. (Note: The more conventional approach, i.e., the prediction of the complex from two separate protein chains, resulted in unconvincing models.)

The models for isolated Connectase (Fig. 1, step 1 and 5), the isolated recognition sequences (step 1, 3 and 5) and the reaction intermediate (N-Cnt, step 3) were predicted with the Colab AlphaFold implementation available under https://colab.research.google.com/github/sokrypton/ColabFold/blob/main/AlphaFold2.ipynb, using standard settings.

## Materials

I freely distribute the materials required to perform the assays in this paper. The kits include Cy5.5-conjugated peptides (100 µg; sufficient for thousands of gels), plasmids for bacterial expression of Connectase, and a reference protein. I appreciate feedback to further improve the method.

### Supplementary Information

Supplementary Information 1.

Supplementary Information 2.

## Data Availability

All primary data are available on the Mendeley public repository (uploaded upon acceptance of the manuscript). All relevant data are also available from the corresponding author upon request.
