# Supplementary material for: Detection and quantification of C-terminally tagged proteins by in-gel fluorescence
Source: Sci Rep. 2024 Jul 8;14:15697. doi: 10.1038/s41598-024-66132-8 (PMC11231263; doi:10.1038/s41598-024-66132-8)
Supplement: Supplementary file 2 — Supplementary Information 2. [file 41598_2024_66132_MOESM2_ESM.pdf]

Detection and quantification of C-terminally tagged proteins by in-gel fluorescence

Supplementary Information

Comparison between Connectase and other protein ligases

A number of enzymes have been used for protein conjugations, including Sortase A, Butelase, Asparaginyl endopeptidase, Trypsiligase, and Subtiligase[1]. All these enzymes belong to the cysteine- or serine protease families, and they catalyze both the intended ligation and the (usually unintended) hydrolysis of their substrates. The ligation reaction is reversible, while the hydrolysis reaction is not. Therefore, the reaction must be stopped at the right time to prevent the complete hydrolysis of educts and products.

The recognition sequences of these enzymes are often short, leading to a low specificity. The most specific representative is Sortase A[2], which catalyzes the fusion of substrates **A** and **B** in form of **A**-LPXTG (X = any amino acid) and G-**B** to yield **A**-LPXTG-**B** (usually with additional linker sequences). This means that it is relatively specific for substrate **A**, but promiscuous towards substrate **B**. Here, the glycine may be replaced by lysine side chains or by other amines[3]. In addition, Sortase A has a low affinity for its substrates and therefore requires high educt concentrations for moderate activity ($K_{M(LPETG)}$ = 7330 µM; $K_{M(GGGGG)}$ = 196 µM)[4]. Although some of these parameters could be improved by the generation of mutant proteins[5,6], they still define the range of potential applications. Sortase is often successfully used to label proteins with peptides, but these experiments are usually conducted with pure educts at a high concentration. This is different from western-blot-like protein detection applications, where small protein quantities must be labeled in a complex solution. Consequently, Sortase is currently not used for such applications (to the best of my knowledge).

Connectase is different from other enzyme ligases. It is not a protease and it does not hydrolyze educts or products[7]. In addition, the recognition sequence is dramatically longer. For example, Connectase from *M. mazei* catalyzes the fusion of **A**-ELASKDPGAFDADPLVVEI and PGAFDADPLVVEI-**B** to form **A**-ELASKDPGAFDADPLVVEI-**B.** The longer recognition sequence comes with a much higher specificity and catalytic efficiency, compared to other protein ligases. It works faster with low substrate concentrations and fuses only substrates carrying the recognition sequence, even in complex solutions. For this reason, this protein ligase is uniquely suited for protein detection applications.

Figures

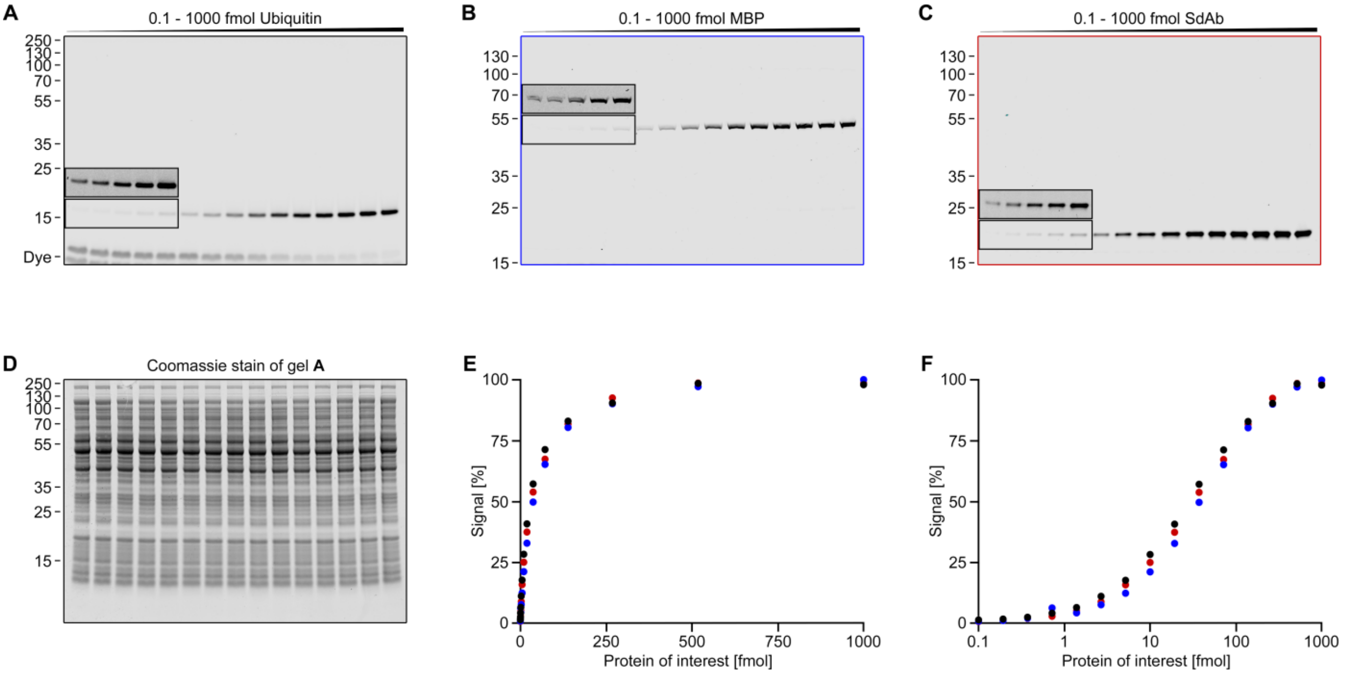

Figure S1: Signal-substrate relationship of in-gel fluorescence assays. A serial dilution of three proteins, Ubiquitin (A), MBP (B), and SdAb (C), was prepared in *E. coli* cell extract (20 µg protein per lane). The proteins were detected via in-gel fluorescence (A-C; the insets show the same area of the gel with increased contrast) and Coomassie stains of the gels prepared (see source data file; D shows one representative gel). The band signals were determined densitometrically and plotted against the protein quantities on a linear (E) or logarithmic (F) scale. Ubiquitin signals are represented as black dots, MBP signals as blue dots, and SdAb signals as red dots.

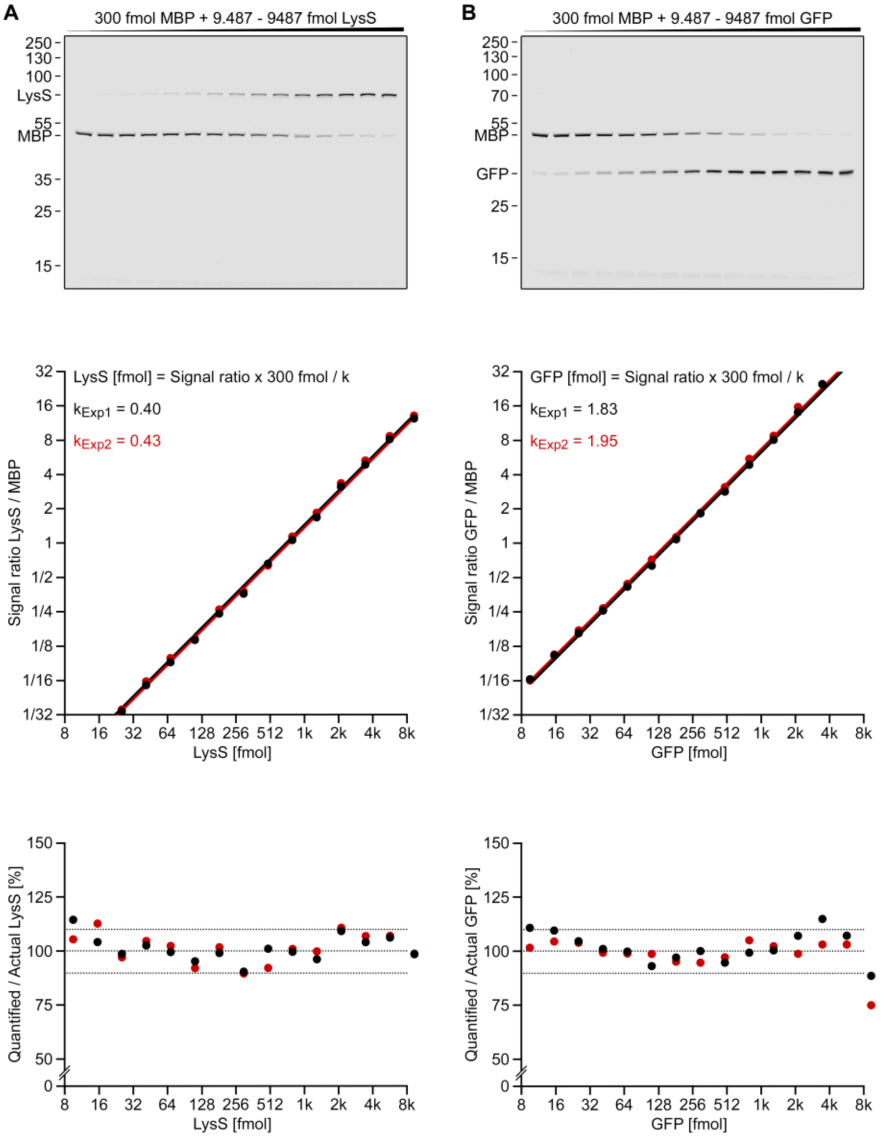

Figure S2: Competition assays with constant reference protein quantities enable accurate POI quantifications. Serial dilutions of two C-terminally tagged proteins, LysS (A) and GFP (B), were prepared in two independent experiments (n = 2). They were mixed with constant quantities of a tagged reference protein (300 fmol MBP) in *E. coli* cell extract (20 µg per lane). The samples were analyzed via in-gel fluorescence (top layer; only one representative gel (Experiment 1) is shown for each POI). The signal ratio (POI signal / reference signal) was determined densitometrically and plotted against the POI quantities (middle layer). For each data series (Exp.1 (black) and Exp. 2 (red)), a linear data fit ($y = k*x$, see Equation 2) with slope k was created. The deviation of the actual data from these data fits are analyzed on a linear y-axis scale in separate plots (bottom layer). Consequently, these plots show which errors are made when quantifying samples using Equation 2 or Equation 3.

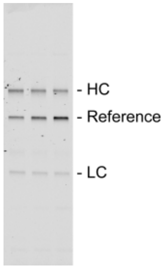

Figure S3: Determination of the $k_{(\alpha HER2/MBP)}$. Tagged αHER2 and MBP were mixed in a 2:1 (lane 1), 1:1 (lane 2), or 1:2 (lane 3) molar ratio. The signal ratio of the bands was determined densitometrically and used to determine $k_{(\alpha HER2/MBP)}$ = 1.03 (see Eq. 4). Note that this value will change slightly with the proportion of expressed heavy and light chains. This effect, however, is small enough to warrant a good estimation of antibody expression levels (Figure 9).

Assay Protocol

For quantitative analysis: sample preparation

(1) Prepare samples containing the C-terminally tagged protein of interest. Include controls to verify the linearity of the assay. Important: All samples should contain the same buffer (or the same quantities of cell extract), the same volume and should be prepared in the same tubes.
(2) Mix 20 µl of each sample to be analyzed with 20 µl reference protein solution (240 nM).
(3) Optional: For absolute quantification, mix 20 µl of a sample of known concentration (240 nM) with 20 µl reference protein solution (240 nM). This sample is used to determine k (see below). Use replicates to increase the accuracy and/or a dilution series to verify the linearity of the assay.

For qualitative and quantitative analyses: Labeling and SDS-PAGE analysis

(1) Prepare a labeling solution containing 40 nM Connectase and 4 nM fluorescent peptide.
(2) Mix 20 µl labeling solution with 40 µl sample (see above: sample preparation).
(3) Incubate for 20 min at room temperature.
(4) Add 20 µl 4x SDS-PAGE loading buffer (optional: heat incubation).
(5) Load 5 µl on an SDS-gel. The rest of the sample can be stored in the freezer and used for future gels.
(6) Run the gel.
(7) Image on a suitable fluorescence imager.
(8) Optional: Store the gel in fixation solution (50% methanol / 10% acetate).

For quantitative analysis: densitometric analysis

(1) Obtain band density values with the instrument software, the free program ImageJ or Image Studio.
(2) In Excel, divide all protein of interest signals by the reference protein signals to obtain the signal ratio.
(3) For relative quantifications, compare the signal ratio of the different samples. A twice as high signal ratio (e.g., 1.5 vs 0.75) signifies twice as much protein of interest.
(4) For absolute quantifications, determine the signal ratio for the sample of known concentration (240 nM). This value is identical with k (see Eq. 4).
(5) Use the relationship $[POI] = \frac{Signal\ ratio\ x\ [Ref]}{\mathrm{k}}$ to determine absolute target protein quantities in each sample.

Notes

Sample preparation. Almost any sample containing tagged POI may be analyzed with the method. This includes cell extracts (Figures 4 and 5) and cell culture medium (Figure 9). Impurities do not affect POI detection. The sample may contain a wide range of POI quantities:

For qualitative analyses:
- at least 0.1 fmol POI (detection limit, Figure 6)
- there is no maximum POI quantity, as signal saturation is prevented by the limited amounts of fluorophore (e.g., Figure 7 shows 10000 fmol protein).
- preferably, >300 fmol should be used, in order to obtain strong bands.

For qualitative analyses:
- One should aim for a signal ratio (POI / Ref) of 0.05 - 20 (Figure 7). With 300 fmol reference protein and k = 1, this corresponds to 15 - 6000 fmol POI.
- Without knowing the k value, estimated POI quantities of roughly 300 fmol should be analyzed. In most cases, this allows quantifications, even when the estimate is wrong by a factor of 10. Where no rough estimates can be made, a given sample should be analyzed in a dilution series (e.g., no dilution, 1:5, 1:25, 1:125).

Some basic rules to estimate POI quantities in unknown samples:
- in order to load 300 fmol POI, 2.5 µl sample with a POI concentration of 120 nM may be used. This corresponds to a POI concentration of 1.8 mg/l for a 30 kDa protein, or a concentration of 6 mg/l for a 100 kDa protein.
- *E. coli* cell cultures contain ~5 x 10^{10} cells at an $OD_{600\ nm}$ = 1, corresponding to a total protein concentration of ~75 mg/l. Here, the average protein (30 kDa, 4288 protein-coding genes) is present at a concentration of ~ 0.525 nM. A protein overexpressed with the T7 promoter/IPTG system may instead be present at a concentration of ~ 563 nM (assuming a 30 kDa protein, making up 25% of the total protein).
- HEK293 cells at 80% confluency have a density of ~6 x 10^4 cells/cm^2. When covered with 100 µl medium (e.g., a T75 flask with 7.5 ml medium), this corresponds to a total protein concentration of ~60 mg/l (assuming a cellular volume of 5 x 10^{-12} l with 200 g/l proteins). Here, the average protein (~40 kDa, ~20000 protein-coding genes) is present at a concentration of ~0.068 nM. An overexpressed protein may instead be present at a concentration of ~ 14 nM (assuming a 40 kDa protein, making up 1% of the total protein).
- The same calculations can be made for other cells. The desired concentration (e.g., 120 nM) can be obtained by centrifugation of the cells.
- The total protein concentration in cell lysates, as described above (e.g., an estimated 75 mg/l for an E. coli culture at OD = 1), can be determined with Bicinchoninic acid or Bradford assays.
- Alternatively, it is always an option to just analyze the highest sample concentration available and a dilution series thereof.

The protein of interest. I typically clone the *M. mazei* Connectase recognition sequence, RELASKDPGAFDADPLVVEI, without additional linker sequence to the C-termini of target proteins. One could consider adding a C-terminal proline to protect against exopeptidases (if that is a problem).

Reference protein. Any well behaved homogeneous tagged protein can be used as a reference protein. For the assays in this study, maltose binding protein was used.

Fluorescent peptide reagent. I have used PGAFDADPLVVEISEEGE-Cy5.5 as a reagent. The type of fluorophore can be varied depending on the instrument for detection. The use of (Infra-)red fluorophores is expected to result in a better signal-to-noise ratio. The peptide sequence is based on the *M. mazei* Connectase (entry mma:MM_2909 in the KEGG database) interaction partner MtrA (MM_1543, residues 155 - 167). If a Connectase variant from a different organism is used, it should be adapted to the respective MtrA sequence of that organism.

Reaction buffer. *M. mazei* Connectase is active over a wide range of pH values and salt concentrations. It tolerates relatively high levels of detergents, DMSO and urea (Fuchs ACD, Nat Commun (2023)). Thus, it is usually not necessary to adapt the sample buffer for qualitative analysis. For comparative or quantitative analyses, it is important to use the same buffer in all samples. For example, if the POI is in cell extract, dilutions should be made with POI-free cell extract. The labeling reagent (see above) should be prepared in detergent-free buffer without impurities (e.g., BSA).

Avoiding sample loss. For quantitative analyses, it is important to avoid sample loss. I recommend the use of PCR tubes or low protein binding tubes. For dilution series, *E. coli* cell extract proved effective for avoiding sample loss.

Residual fluorescent peptide. Residual fluorescent peptide can be detected as an extra band slightly above the level of the bromophenol blue dye front. This can be avoided by running the gels slightly longer. In presence of cell extract, this band may disappear in the course of the labeling reaction, possibly because of peptide-degrading proteases.

Fluorophore-induced band shift. The labeling with fluorophore increases the size of the target protein by ~1 kDa and therefore results in a small band shift.

Comparability of signals on different gels. In contrast to Western blots, technical replicates of quantitative in-gel fluorescence competition assays produce almost identical signal ratio data (not raw signals). Therefore, signal ratios on different gels may be compared if all assay parameters are kept identical. Nevertheless, it is good practice to include controls on each gel. For example, the same sample could be included in two series of experiments (i.e. labeling reactions) that should be compared. Note that signal ratios cannot be compared between different preparations of the same protein. In these cases, it must first be validated that the samples behave the same way.

Connectase from different organisms. I have chosen *M. mazei* Connectase, because it expresses well, is reasonably stable and soluble at high concentrations, and has an activity optimum at neutral pH and moderate salt concentrations. It is likely, however, that more favorable Connectase homologs exist among the highly diverse range of sequences. These homologs may recognize mutually exclusive recognition sequences, allowing two separate labeling reactions with different fluorophores in parallel (multiplexing).

Storing the SDS-gel. The gel should be stored in fixation solution (50% methanol / 10% acetate). The Cy5.5 fluorophore employed in this paper is not particularly light sensitive. However, for longer storage (several days), the gel box should be wrapped in aluminium foil and stored at low temperatures. The signal is then stable for weeks, although small proteins tend to diffuse.

Sharp bands. Quantitative analyses are more accurate with sharper gel bands. I therefore recommend the use of commercial polyacrylamide gels and the application of low and identical volumes (e.g., 5 µl) of sample.

References

1 Schmidt, M., Toplak, A., Quaedflieg, P. J. & Nuijens, T. Enzyme-mediated ligation technologies for peptides and proteins. *Curr Opin Chem Biol* **38**, 1-7, doi:10.1016/j.cbpa.2017.01.017 (2017).

2 Pishesha, N., Ingram, J. R. & Ploegh, H. L. Sortase A: A Model for Transpeptidation and Its Biological Applications. *Annu Rev Cell Dev Biol* **34**, 163-188, doi:10.1146/annurev-cellbio-100617-062527 (2018).

3 Heck, T., Pham, P. H., Yerlikaya, A., Thony-Meyer, L. & Richter, M. Sortase A catalyzed reaction pathways: a comparative study with six SrtA variants. *Catal Sci Technol* **4**, 2946-2956, doi:10.1039/c4cy00347k (2014).

4 Frankel, B. A., Kruger, R. G., Robinson, D. E., Kelleher, N. L. & McCafferty, D. G. Staphylococcus aureus sortase transpeptidase SrtA: insight into the kinetic mechanism and evidence for a reverse protonation catalytic mechanism. *Biochemistry* **44**, 11188-11200, doi:10.1021/bi050141j (2005).

5 Chen, L. *et al.* Improved variants of SrtA for site-specific conjugation on antibodies and proteins with high efficiency. *Sci Rep* **6**, 31899, doi:10.1038/srep31899 (2016).

6 Jacobitz, A. W., Kattke, M. D., Wereszczynski, J. & Clubb, R. T. Sortase Transpeptidases: Structural Biology and Catalytic Mechanism. *Adv Protein Chem Str* **109**, 223-264, doi:10.1016/bs.apcsb.2017.04.008 (2017).

7 Fuchs, A. C. D. *et al.* Archaeal Connectase is a specific and efficient protein ligase related to proteasome β subunits. *Proceedings of the National Academy of Sciences* **118**, e2017871118, doi:10.1073/pnas.2017871118 (2021).
